# Reply to Engelthaler and Meyer, “Furthering the Continental Drift Speciation Hypothesis in the Pathogenic *Cryptococcus* Species Complexes”

**DOI:** 10.1128/mSphere.00242-17

**Published:** 2017-06-14

**Authors:** Arturo Casadevall, Joudeh B. Freij, Christopher Hann-Soden, John Taylor

**Affiliations:** a Department of Molecular Microbiology and Immunology, Johns Hopkins Bloomberg School of Public Health, Baltimore, Maryland, USA; b University of California—Berkeley, Berkeley, California, USA; Duke University Medical Center

## REPLY

We thank and compliment Engelthaler and Meyer for their letter (1), which provides new information extending our hypothesis that continental drift was a major influence in driving speciation within the *Cryptococcus* species complexes (2). Their map superimposing the four major current *Cryptococcus gattii* molecular types/species (VGI, VGII, VGIII, and VGIV) on the map of Pangea sheds light on possible origins of Asian, Indian, and Iberian isolates and clearly delineates areas that are unexplored and in need of sampling. We are encouraged by the notion that viewing cryptococcal speciation in the larger context of continental drift is already stimulating new thought, and we hope that it also catalyzes new experimental work to separate ancient speciation events in deep geologic time from more recent introductions of isolates in defined geographic areas.
